# Human papillomavirus vaccination and all-cause morbidity in adolescent girls: a cohort study of absence from school due to illness

**DOI:** 10.1093/ije/dyab003

**Published:** 2021-02-06

**Authors:** Anders Hviid, Nicklas M Thorsen, Louise N Thomsen, Frederik T Møller, Andreas Wiwe, Morten Frisch, Palle Valentiner-Branth, Dorte Rytter, Kåre Mølbak

**Affiliations:** 1 Department of Epidemiology Research, Statens Serum Institut, Copenhagen, Denmark; 2 Section for Epidemiology, Department of Public Health, Aarhus University, Aarhus, Denmark; 3 Division of Infectious Diseases Preparedness, Statens Serum Institut, Copenhagen, Denmark; 4 Ledelsesinformation, data og analyser, Børne- og Ungdomsforvaltningen, City of Copenhagen, Denmark; 5 Department of Clinical Medicine, Center for Sexology Research, Aalborg University, Aalborg, Denmark; 6 Department of Veterinary and Animal Sciences, University of Copenhagen, Frederiksberg C, Denmark

**Keywords:** HPV vaccination, vaccine safety, school absence, all-cause morbidity

## Abstract

**Background:**

A growing body of evidence supports the safety of the human papillomavirus (HPV) vaccines. However, concerns about autonomic dysfunction syndromes and non-specific symptoms continue to linger. These conditions are not easily captured by traditional diagnostic classification schemes and call for innovative approaches to the study of vaccine safety which take more general measures of all-cause morbidity into account.

**Methods:**

Taking advantage of the unique Danish registers, including regional registration of absence from school, we conducted a cohort study of 14 068 adolescent Danish girls attending 5th through 9th grade in public schools in the municipality of Copenhagen during 1 August 2013–23 January 2018. We obtained time-varying HPV vaccination status and demographic information from nationwide registers. Using Poisson regression with random effects, we estimated rate ratios (RRs) of absence due to illness, comparing HPV-vaccinated girls with unvaccinated girls with adjustment for grade, season, calendar period, demographic factors and random effects at the individual, class and school levels.

**Results:**

Our study included 6 206 188 school days with 213 221 days of absence from school due to illness (absence rate, 3.4%). Comparing absence rates due to illness in HPV-vaccinated and unvaccinated girls yielded an adjusted RR of 1.00 (95% confidence interval [CI], 0.98–1.03).

**Conclusions:**

Our study provides an important and novel contribution to HPV vaccine safety. Using absence from school records, we were able to address important safety concerns without relying on medical diagnoses. We conclude that HPV vaccination does not increase the risk of morbidity in any manner that manifests as absence from school due to illness.

**Key Messages**

- A regional Danish register of school absence due to illness has provided a unique opportunity to study human papillomavirus (HPV) vaccine safety concerns that cannot easily be studied using traditional diagnostic codes.
- In a regional Danish cohort of 14 068 adolescent girls, HPV-vaccinated girls and unvaccinated girls had virtually identical rates of absence from school due to illness.
- Our study provides a strong argument that HPV vaccination does not increase the risk of any forms of illness, including illness which is not recorded in administrative registers using diagnostic classification schemes.

## Introduction

Human papillomavirus (HPV) vaccines have now been in use for more than a decade. Despite an excellent safety record demonstrated in numerous studies,^1^ concerns about adverse events in the form of autonomic dysfunction syndromes continue to linger and have caused serious setbacks in national immunization programmes in countries such as Japan, Denmark and Ireland. Most of these concerns have originated from case reports and sensationalist media reporting, and have not been substantiated in observational studies.^2–5^ A closer look at the spontaneous adverse event reports have revealed that specific syndromes are rare in contrast to non-specific symptoms such as fatigue, headache, nausea and dizziness. In the US Vaccine Adverse Event Reporting System, only one case of postural orthostatic tachycardia syndrome (POTS) was reported per 6.5 million doses of distributed HPV vaccine.^6^ In contrast, headache, dizziness, fatigue and nausea were common among reports. In our own evaluation of HPV vaccine adverse event reports in Denmark, fatigue, headache and dizziness were the three most commonly reported symptoms.^7^ Non-specific symptoms are obviously difficult to study using databases and registers, in contrast to outcomes with straightforward diagnostic guidelines. These difficulties call for new and innovative approaches to measure the impact of vaccination on all-cause morbidity, not easily captured through studies relying on diagnostic classification systems. We took advantage of the unique nationwide registers in Denmark and conducted a pioneering study of the association between HPV vaccination and absence from school due to illness, in a large cohort of Danish children. We hypothesized that if HPV vaccination is associated with increased risk of non-specific symptoms among HPV-vaccinated girls, this would result in more absence from school due to illness than among their unvaccinated peers.

## Methods

### Study cohort and absence from school

We constructed a study cohort from a regional registration system of school attendance records from the municipality of Copenhagen—the capital of Denmark. This registration covered all public schools in Copenhagen from the start of the school year 2013/2014 (1 August 2013) to 23 January 2018, and contained daily attendance records for individual children with information on school, grade and class. In the register, possible absence for each school day was recorded as absence due to illness, exceptional absence or illegal absence. We included girls in grades 5–9. Each girl was identifiable through a unique personal ID assigned to all people living in Denmark.^8^ This unique ID is used in all other national registers and allows for individual-level linkage of health-related information, including vaccinations, and demographic information. The study was approved by the Danish Data Protection Agency (approval no. 19/07650). Ethical approval is not needed for register-based research in Denmark.

### HPV vaccination and demographic information

The Danish childhood vaccination programme is voluntary and free of charge. HPV vaccination with a quadrivalent vaccine (Gardasil^®^, MSD) was included in the Danish schedule for 12-year-old girls on 1 January 2009, with catch-up vaccination of 13–15 year old girls starting October 2008. The bivalent vaccine (Cervarix^®^, GSK) replaced the quadrivalent vaccine between 1 February 2016 and 31 October 2017. Since 1 November 2017, the nonavalent vaccine (Gardasil9^®^, MSD) has been used. We obtained information on HPV vaccinations from the Danish Vaccination Registry, which contains individual-level longitudinal information on all vaccinations in the Danish childhood vaccination programme.^9^

We obtained information on birth order, the mother’s age at the time of birth and the mother’s civil status, from the Danish Civil Registration System for each girl in the study cohort.^8^

### Statistical methods

In our main analysis, we analysed days of absence due to illness during the study period according to HPV vaccination status and other study covariates (Supplementary Materials—Description of data, available as Supplementary data at *IJE* online). Vaccination was considered a time-varying variable. To take individual-level effects and cluster effects from schools and classes into account, we used generalized linear mixed effects modelling (Supplementary Materials—Generalized linear mixed effects models, available as Supplementary data at *IJE* online) in the form of Poisson regression with random effects to estimate the effect of vaccination on absence from school due to illness, using rate ratios (RRs) comparing absence rates in HPV-vaccinated and unvaccinated girls.^10^ Unless otherwise specified, all vaccination effects were estimated with adjustment (adjusted RRs) for calendar year (2013–2018 in 1-year intervals), season (January-March, April-June, July-September, October-December), grade (5th , 6th , 7th, 8th , 9th), birth order (1, 2, 3, 4+), age of the mother at the time of birth (up to 19 years, 20–24, 25–29, 30–34, 35–39, 40+ years) and the civil status of the mother at baseline (married, unmarried, divorced or dead) as fixed effects, while handling over-dispersion through random effects at the individual, class and school levels. Random effects were modelled as random intercepts. Class was considered a nested random effect inside school, whereas the individual effect was not, due to the possibility of girls changing schools during the study period. A class was defined as a given class at a given school within a given year. In secondary analyses, we estimated RRs stratified according to: (i) calendar year of first vaccination (before 2011, 2012–2016 in 1-year intervals, 2017+), grade at first vaccination (before study entry, 5th , 6th , 7th , 8th , 9th) and season at first vaccination (January-March, April-June, July-September, October-December); and (ii) time since the most recent HPV vaccination (0–13 days, 14–90 days, 91–365 days, 366+ days).

To evaluate the association between vaccination and longer periods of absence due to illness, we analysed: (i) days of absence due to illness in excess of a certain number of days; and (ii) periods of absence due to illness of at least a given length (Supplementary Materials—Description of data). Similar to the main analysis of single days of absence, we used Poisson regression with random effects to estimate adjusted RRs comparing rates of excess days or rates of periods in HPV-vaccinated and unvaccinated girls. We considered periods of length 1 to 21 days (in 1-day increments) and days in excess of 0 to 20 days (in 1-day increments). These datasets become increasingly sparse with increasing period lengths, resulting in increasing levels of over-dispersion and convergence issues. We evaluated the robustness of the Poisson regression with random effects by using perturbation analysis (Supplementary Materials—Robustness, available as Supplementary data at *IJE* online).^11^

We conducted a sensitivity analysis of absence due to illness, where we only included girls who were HPV-unvaccinated at entry into the study: a ‘new vaccinee’ design. We further conducted an analysis of all days of absence in contrast to only absence due to illness, and repeated the main analysis of absence due to illness using negative binomial regression with random effects. Finally, we modelled the impact of misclassification of truancy as absence due to illness, and the sensitivity of our approach when associations between HPV vaccination and cause-specific morbidity were simulated in our data (Supplementary Materials—Modelling the impact of bias, available as Supplementary data at *IJE* online).

Data management and statistical analyses were conducted using R version 3.5.1 [R Core Team (2017)]. Mixed effects modelling was conducted using the *lme4* package. Perturbation analysis was conducted using the *perturb* package.

## Results

**Figure 1:**
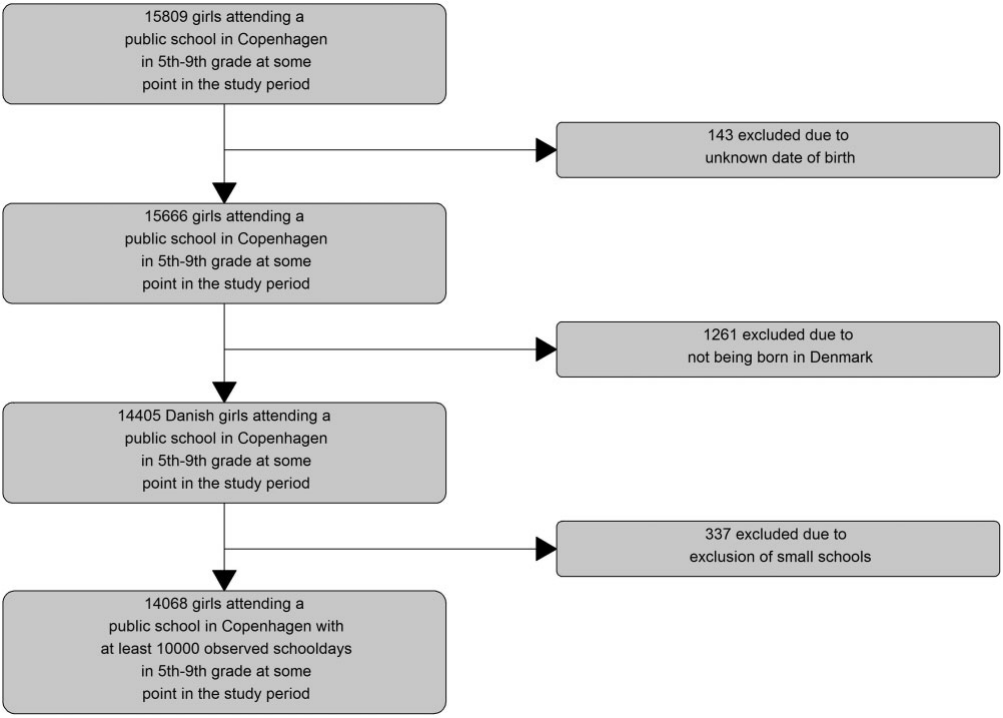
Study flowchart

The Copenhagen municipality registration system of school attendance comprised attendance records for 6 786 767 school days in the 1 August 2013–23 January 2018 period for 15 809 girls attending 5^th^-9th grade. We excluded 143 girls with unknown date of birth or invalid ID, 1261 girls who were not born in Denmark and 337 girls attending schools with less than 10 000 registered school days in the study period (see study flowchart in Figure 1). This resulted in 14 068 girls in the final study cohort with 6 206 188 school days during follow-up (mean number of school days of follow-up, 441.2 days). The study cohort comprised records from 60 unique schools and 3542 unique classes. There were: 480 934 days of absence in the study (absence rate, 7.7%); 113 830 days of exceptional absence (1.8%); 153 883 days of illegal absence (2.5%); and 213 221 days of absence due to illness (3.4%). Absence due to illness increased slightly with increasing grade (absence rate of 3.2% in 5th grade compared with 3.5% in 9th grade), was fairly consistent across calendar years except for 2018 (3.2%) and was highest in the January-March period (4.3%) (Table 1). Girls with older mothers (40+ years of age, 4.1%), without older siblings (3.5%) and with unmarried, divorced or dead mothers (3.9%) had the highest absence rates (Table 1). The girls in the study spent 3 432 675 days as HPV-vaccinated and 2 773 513 as unvaccinated with similar absence rates in the two periods (3.4% and 3.5% for vaccinated and unvaccinated girls, respectively)(Table 1).

**Table 1:** Descriptive characteristics of 14 068 girls attending 5^th^-9th grade in public schools (Copenhagen, Denmark) during 1 August 2013–23 January 2018

Characteristic	Number of girls	Human papillomavirus vaccinated school days of follow-up [100 000 days] (illness absence rate)	Unvaccinated school days of follow-up [100 000 days] (illness absence rate)	All school days of follow-up [100 000 days] (illness absence rate)
Total	14 068	34.3 (3.4%)	27.7 (3.5%)	62.1 (3.4%)
Grade				
5th	7964	0.6 (3.0%)	13.4 (3.2%)	13.9 (3.2%)
6th	7396	5.2 (3.1%)	7.7 (3.4%)	12.9 (3.3%)
7th	7135	8.7 (3.3%)	3.7 (4.2%)	12.5 (3.6%)
8th	6782	10.0 (3.5%)	1.9 (4.1%)	11.8 (3.6%)
9th	6189	9.8 (3.4%)	1.1 (4.5%)	10.9 (3.5%)
Calendar year				
2013	6766	3.8 (3.3%)	2.2 (3.4%)	6.0 (3.4%)
2014	8450	8.8 (3.3%)	4.6 (3.4%)	13.4 (3.4%)
2015	8570	8.1 (3.4%)	5.4 (3.5%)	13.6 (3.4%)
2016	8743	6.9 (3.3%)	7.2 (3.6%)	14.1 (3.5%)
2017	8919	6.3 (3.5%)	7.8 (3.6%)	14.1 (3.5%)
2018	7259	0.4 (3.2%)	0.5 (3.1%)	0.9 (3.2%)
Season				
January-March	13 823	9.3 (4.3%)	6.9 (4.4%)	16.2 (4.3%)
April-June	11 925	9.0 (2.5%)	5.5 (2.9%)	14.4 (2.7%)
July-September	13 869	6.5 (2.9%)	6.7 (2.8%)	13.2 (2.9%)
October-December	13 870	9.6 (3.5%)	8.7 (3.7%)	18.2 (3.6%)
Mother’s age at birth				
up to 19 years	228	0.5 (2.7%)	0.4 (3.1%)	0.9 (2.8%)
20-24	1775	4.8 (3.2%)	3.1 (3.7%)	7.9 (3.4%)
25-29	4427	11.4 (3.4%)	8.7 (3.4%)	20.1 (3.4%)
30-34	4935	11.5 (3.3%)	10.0 (3.5%)	21.4 (3.4%)
35-39	2246	5.2 (3.6%)	4.6 (3.6%)	9.8 (3.6%)
40 years +	457	0.9 (3.6%)	1.0 (4.4%)	1.9 (4.1%)
Birth order				
1	6821	16.0 (3.4%)	13.8 (3.5%)	29.8 (3.5%)
2	4677	11.7 (3.5%)	9.0 (3.6%)	20.8 (3.5%)
3	1648	4.1 (3.0%)	3.1 (3.4%)	7.3 (3.2%)
4+	922	2.4 (3.0%)	1.8 (3.3%)	4.2 (3.2%)
Mother’s civil status				
Married	7704	19.0 (2.9%)	15.0 (3.2%)	34.1 (3.0%)
Unmarried, divorced or dead	6364	15.3 (3.9%)	12.7 (4.0%)	28.0 (3.9%)

Comparing absence rates due to illness in HPV-vaccinated and unvaccinated girls yielded an adjusted RR of 1.00 [95% confidence interval (CI), 0.98–1.03] (Table 2). Tests of homogeneity for the stratifying variables time since last vaccination, grade at first vaccination, year of first vaccination and season at first vaccination were all highly significant (p ≤ 0.001, Table 2). However, this primarily reflected the study’s high statistical power, and none of the few significantly increased estimates were more than 5% increased (Table 2).

**Table 2:** Rate ratios (RRs) of absence from school due to illness according to human papillomavirus (HPV) vaccination status among 14 068 girls attending 5^th^-9th grade in public schools (Copenhagen, Denmark) during 1 August 2013–23 January 2018

HPV vaccination status	Number of days absent due to illness (school days of follow-up [100 000 days])	RR (95% CI)^a^	*P*-value^b^
Unvaccinated	97 638 (27.7)	1 (reference)	–
Vaccinated	115 583 (34.3)	1.00 (0.98-1.03)	0.71
Time since last vaccination:^c^			<0.001
0-13 days	2321 (0.7)	1.01 (0.96-1.05)	
14-90 days	11 553 (3.6)	1.03 (1.00-1.05)	
91-365 days	27 431 (8.5)	0.98 (0.96-1.00)	
366+ days	74 278 (21.5)	1.02 (0.99-1.05)	
Grade at first vaccination:^c^			<0.001
Before study entry	65 916 (19.0)	0.93 (0.88-0.98)	
5^th^ grade	13 436 (4.2)	1.04 (1.00-1.09)	
6^th^ grade	30 522 (9.5)	1.00 (0.98-1.03)	
7^th^ grade	4371 (1.3)	1.03 (0.98-1.08)	
8^th^ grade	1194 (0.4)	0.97 (0.90-1.05)	
9^th^ grade	144 (0.1)	0.68 (0.55-0.83)	
Year of first vaccination:^c^			<0.001
2010 and before	5295 (1.7)	0.76 (0.67-0.85)	
2011	16 139 (4.5)	0.81 (0.74-0.89)	
2012	23 845 (7.0)	0.85 (0.79-0.91)	
2013	26 423 (7.9)	0.95 (0.90-1.00)	
2014	22 393 (6.5)	1.03 (0.99-1.07)	
2015	11 260 (3.5)	1.01 (0.97-1.05)	
2016	5622 (1.8)	1.01 (0.97-1.05)	
2017 and after	4606 (1.5)	1.02 (0.98-1.06)	
Season at first vaccination^c^			0.001
January-March	26 267 (8.2)	0.97 (0.94-1.00)	
April-June	34 166 (9.9)	1.04 (1.00-1.07)	
July-September	27 323 (8.1)	0.98 (0.95-1.01)	
October-December	27 827 (8.2)	1.03 (0.99-1.06)	

a Adjusted for calendar year (2013-18 in 1-year intervals), season (January-March, April--June, July-September, October-December), grade (5th, 6th, 7th, 8th, 9th), birth order (1, 2, 3, 4+), age of the mother at the time of birth (up to 19, 20–24, 25–29, 30–34, 35–39, 40+ years), the civil status of the mother at baseline (married, unmarried, divorced or dead) and random effects at the individual, class and school levels.

b Test for homogeneity.

c Reference category is ‘Unvaccinated’.

**Figure 2:**
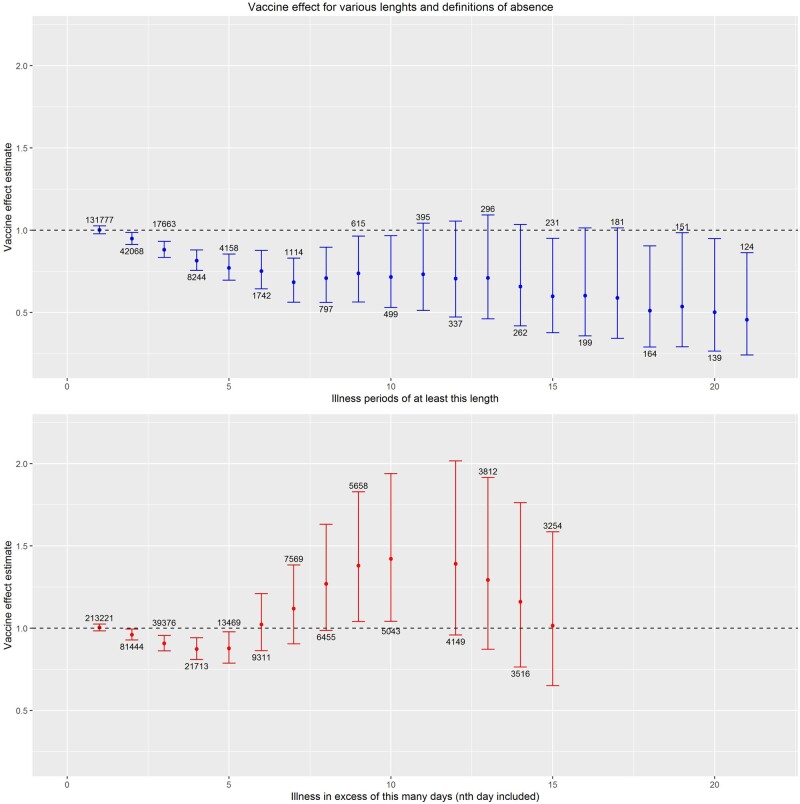
(A) Rate ratios (y-axis) (with 95% confidence interval bars) of periods of absence from school due to illness according to human papillomavirus vaccination status among 14 068 Danish girls attending 5^th^-9th grade during 1 August 2013–23 January 2018. Periods (x-axis) defined as at least x consecutive days; that is, x = 5 refers to all periods spanning 5 or more days of consecutive absence due to illness. Numbers represent the total number of periods in the analysis. (B) Rate ratios (y-axis) (with 95% confidence interval bars) of days of absence from school due to illness according to human papillomavirus vaccination status among 14 068 Danish girls attending 5th-9th grade during 1 August 2013–23 January 2018. Days (x-axis) defined as all days in excess of x days in a period; that is, x = 5 refers to the number of days in excess of 5 days in periods spanning 5 or more days. Numbers represent the total number of days in the analysis. Missing estimates correspond to regression models which did not converge (see Supplementary Materials for further details, available as Supplementary data at *IJE* online)

The majority of absence periods in our study were of short duration. Among 213 221 days of absence in the study, only 9311 days (4.4%) occurred after 1 week of absence (5 school days) (Figure 2). Similarly, among 131 777 periods of any length, only 1742 (1.3%) were longer than 1 week (Figure 2). HPV vaccination was not associated with increased rates of any absence periods; the adjusted RR for periods of more than 1 week was 0.75 (95% CI, 0.64–0.88) and 0.73 (95% CI, 0.51–1.04) for periods of more than 2 weeks (Figure 2). When analysing excess days of absence, the adjusted RR for days occurring after 1 week of absence was 1.02 (95% CI, 0.86–1.21) (Figure 2). The model for excess days after 2 weeks of absence did not converge (see Supplementary Materials—Robustness, for a discussion of convergence issues in the analyses of excess days); the adjusted RR for excess days after 11 days was 1.39 (95% CI, 0.96–2.02).

Only including girls who were HPV-unvaccinated at entry into the study (*n* = 9412), yielded an adjusted RR of 1.00 (95% CI, 0.98–1.02). Comparing absence rates due to all causes yielded an adjusted RR of 0.98 (95% CI, 0.96–0.99). Using Poisson regression with neither fixed nor random effects yielded a crude RR of 0.96 (95% CI, 0.95–0.96). The negative binomial regression with random effects yielded an RR of 0.97 (95% CI, 0.95–1.00).

Modelling the impact of non-differential misclassification of 5%, 10% and 20% of all single days of absence in the study yielded RRs of 1.00 (95% CI, 0.98–1.03), 1.01 (95% CI, 0.98–1.03) and 1.00 (95% CI, 0.98–1.03), respectively (Supplementary Materials—Modelling the impact of bias). When simulating cause-specific morbidity, we observed that cause-specific morbidity base rates as low as 0.5% (corresponding to an average of a little less than 1 day of absence due to that particular illness per year per girl), would result in statistically significant all-cause morbidity RRs for even modest associations between HPV vaccination and cause-specific morbidity. Even rarer illnesses would also result in significant all-cause morbidity RRs if the association with HPV vaccination was sufficiently strong (Supplementary Materials—Modelling the impact of bias).

## Discussion

We found no support for the hypothesis of HPV vaccination increasing the risk of all-cause morbidity measured as days of absence from school due to illness. This finding was consistent in periods following vaccination, was independent of when vaccination was initiated (grade, calendar year and season) and extended to longer periods of absence.

Studies evaluating the association between HPV vaccination and all-cause morbidity are rare. However, a large body of evidence supporting the safety of HPV vaccination with respect to specific adverse events does exist. In a review of 23 HPV vaccine trials evaluating safety, no increased risk of serious adverse events was reported, RR 0.98 (95% CI, 0.92–1.05).^12^ A number of post-licensure safety evaluations have evaluated a wide range of well-defined clinical outcomes such as autoimmune diseases, neurological conditions and venous thromboembolisms with reassuring results.^5^^,^^13^^,^^14^ Similarly, no well-controlled study has linked HPV vaccination and autonomic dysfunction syndromes in the form of POTS, complex regional pain syndrome or chronic fatigue syndrome.^2–6^

Infectious diseases are common in childhood and likely a major source of absence from school. Among 598 553 Danish children born in Denmark 1987–97, 6.8% had an infectious disease hospital contact from 10 years of age and until completion of the 9th grade, and 27.8% had three or more prescriptions for anti-infective agents.^15^ Another major source of absence is non-specific symptoms related to general health well-being and mental well-being, which are particularly common in adolescent girls according to *Health Behaviour in School-aged Children—a World Health Organization Collaborative Cross-national Study*.^16^ In the most recent Danish report from 2018, 1827 girls participated. Among 13-year-old girls, only 24% considered themselves to be in ‘really good health’; 37% reported weekly headaches; 20% stomach aches; 27% back pain; 24% dizziness; 36% episodes of feeling sad; 48% episodes of irritability; 42% episodes of nervousness; and 45% episodes of sleeping difficulty.^17^ Any evaluation of vaccine safety concerns relying on self-reports of such highly prevalent non-specific symptoms would have to consider the possibility that some vaccinees may have inappropriately attributed their symptoms to vaccination. To the extent such symptoms result in absence from school, our study effectively eliminated this potential source of reporting bias.

Our study has a number of limitations. We cannot verify the validity of absence registered as due to illness. However, classification of truancy as illness is unlikely to be associated with HPV vaccination status and will result in non-differential misclassification. Furthermore, any truancy will most likely occur as single days of absence or very short periods of absence. We observed no consistently increased risks for longer periods, and modelling realistic levels of non-differential misclassification of single days revealed no material impact. Since our study only comprises school days, we have no information on illness during weekends and school holidays. Other than a slight loss in statistical power, this will primarily result in under-reporting of illness period lengths and excess days of illness. Time-dependent healthy vaccinee bias, whereby sick girls avoid or postpone vaccination, can bias results towards a protective effect of vaccination especially in the immediate periods following vaccination. However, there was no protective effect of vaccination on absence in the first 2 weeks after vaccination, suggesting that healthy vaccinee bias, if present, has had little influence on our results, or conversely that healthy vaccinee bias is masking an increased risk of vaccination. In any case, this is unlikely to have any impact on our main results, since absence in the first 2 weeks following vaccination comprises only 1.1% of the total number of days of absence due to illness in our study. We cannot exclude that confounding by lifestyle factors and socioeconomic factors has masked an increased risk associated with vaccination; lifestyle-related and socioeconomic factors might be associated with higher HPV vaccine uptake and might also be associated with better baseline health. However, our analytical approach did include random effects at the individual, class and school levels, which minimizes concern about confounding since the extent to which lifestyle and socioeconomic factors are associated with absence due to illness is addressed indirectly by the random effects. Since this is a study of all-cause morbidity, we cannot exclude that HPV vaccination increases the risk of specific morbidities. However, since our study has a very high degree of statistical power, we can conclude that any such association could only remain undetected in our results if sufficiently rare or for risk increases below clinically significant levels.

Our study only included public schools. However, in the school year 2013/14, 79% of all Danish children attended public schools.^18^ The Copenhagen registration system of absence from school records has not been validated. The overall absence rates due to all causes in our study was 7.7%, with 3.4% being due to illness for 5^th^-9th grade, which corresponds well to the national average in the 2017/18 school year of 5.9% with 3.0% being due to illness for 0-9th grade.^19^ The Danish absence rates compare well with other countries; in England, an absence rate due to illness of 2.6% was reported for the 2017/18 school year for state-funded primary, secondary and special schools.^20^

The major strength of our study is that it captures all morbidity sufficient to cause absence from school, including morbidity not recorded elsewhere. Further strengths include the independent ascertainment of exposure and outcome in a prospective regional cohort and the use of a random effects model taking morbidity patterns at the individual, class and school levels into account.

When introducing a vaccine in a new population, the occurrence of temporal safety signals is to be expected.^21^^,^^22^ Subsequently, these signals must be evaluated in well-controlled observational studies. Our study is the first to address HPV vaccine safety signals comprising morbidity not easily captured in traditional diagnostic classification schemes, and our study provides an important and novel contribution to the science of HPV vaccination safety. We conclude that HPV vaccination does not increase the risk of all-cause morbidity in any manner that manifests as absence from school due to illness, and this is supported by the high statistical power of our study, which allows us to exclude the existence of even minute risk increases in absence due to illness (>3% increase).

The data underlying this article comprise individual-level information on health events and cannot be shared publicly due to privacy concerns.

## Supplementary data

Supplementary data are available at *IJE* online.

## Funding

The study was supported by grants from the Danish Medicines Agency and the Danish Cancer Society. A.H. is supported by a grant from the Novo Nordisk Foundation. The funding bodies had no role in the study design; the collection, analysis and interpretation of the data; the writing of the manuscript; or the decision to submit it for publication. All authors are independent from the funding agencies.

## Supplementary Material

dyab003_Supplementary_Data
