# Supplementary material for: Human papillomavirus vaccination and all-cause morbidity in adolescent girls: a cohort study of absence from school due to illness
Source: Int J Epidemiol. 2021 Feb 6;50(2):518–26. doi: 10.1093/ije/dyab003 (PMC8128452; doi:10.1093/ije/dyab003)
Supplement: dyab003_Supplementary_Data [file dyab003_supplementary_data.zip › ije-2019-12-1617-File005.docx]

**SUPPLEMENTARY** **MATERIALS**

- **Description of data**, pages 2-3.
- **Generalized linear mixed effects models**, pages 4-6.
- **Robustness**, page 7.
- **Modelling the impact of bias and confounding**, pages 8-9.
- **Supplementary Figure S1 Label**, page 10.
- **Supplementary Figure S1**: Distributions of HPV vaccine effect estimates from perturbation analyses.

**Description of School Attendance Records.**

The raw data obtained from the regional registration system of school attendance records from the municipality of Copenhagen comprises single school days for each girl as rows. Each row has a unique ID identifying the girl, date of the day, status of the day (present, exceptional absence, illegal absence, absence due to illness), grade, class and school. We calculate *absence rates* as *number of days absent / total number of days under observation*. In the analyses of periods of absence, we define a period of absence as consecutive days of absence. We calculate *absence period rates* for periods of length *l* as *number of periods of at least length l / total number of days under observation*. For the purpose of allocation of period rates to grade, year, and season in stratified analyses, the *l*th day in a period is used. In the analysis of excess days of absence*,* we calculate *excess days of absence rates* for a period of length *l* as *all consecutive days following the lth day in all periods of at least length l / total number of days under observation.* Both periods and excess days overlapping weekends and holidays (not school days) were considered consecutive. In the following we demonstrate the different ways of counting absence in our study. The table below gives an example of what four school weeks for a given student might look like and how we would count different types of illness-related absence. In the table below, we use P for present, I for ill, and A for absence for any other reasons than illness.

| **Weekday** | **School presence** | **Illness in excess of 2 days** | **Illness periods of at least 2 days** | **Illness in excess of 5 days** | **Illness periods of at least 5 days** |
| --- | --- | --- | --- | --- | --- |
| **Monday** | P | 0 | 0 | 0 | 0 |
| **Tuesday** | I | 0 | 0 | 0 | 0 |
| **Wednesday** | I | 0 | 1 | 0 | 0 |
| **Thursday** | I | 1 | 0 | 0 | 0 |
| **Friday** | P | 0 | 0 | 0 | 0 |
| **Monday** | P | 0 | 0 | 0 | 0 |
| **Tuesday** | A | 0 | 0 | 0 | 0 |
| **Wednesday** | A | 0 | 0 | 0 | 0 |
| **Thursday** | P | 0 | 0 | 0 | 0 |
| **Friday** | P | 0 | 0 | 0 | 0 |
| **Monday** | P | 0 | 0 | 0 | 0 |
| **Tuesday** | I | 0 | 0 | 0 | 0 |
| **Wednesday** | I | 0 | 1 | 0 | 0 |
| **Thursday** | I | 1 | 0 | 0 | 0 |
| **Friday** | I | 1 | 0 | 0 | 0 |
| **Monday** | I | 1 | 0 | 0 | 1 |
| **Tuesday** | I | 1 | 0 | 1 | 0 |
| **Wednesday** | P | 0 | 0 | 0 | 0 |
| **Thursday** | P | 0 | 0 | 0 | 0 |
| **Friday** | P | 0 | 0 | 0 | 0 |

For illness in excess of 2 days we count 5 days of illness-related absence. For illness periods of at least 2 days we count 2 periods. For illness in excess of 5 days we count 1 day. For illness periods of at least 5 days we count 1 period. For all types of absence rates we count 20 schooldays in the denominator.

**Generalized linear mixed effects models**

Generalized linear mixed effects models (GLMMs) extend the generalized linear model to include both fixed and random effects as explanatory variables. An explanatory variable is considered a fixed effect if it is assumed constant for the statistical units of observation. Conversely, it is considered a random effect if it is assumed to be drawn from an underlying distribution. As an example, if school is a fixed effect, then we would estimate the particular effects of attending a given school and get a parameter estimate for each school. On the other hand if school is considered a random effect, the intercept in the model will be for an average school and we will instead get an estimate for the variance of the school random effect summarizing how big the differences are between different schools. GLMMs excel in the analysis of data with hierarchical clusters of non-independent measurements. This has a number of obvious benefits. GLMMs explicitly model non-independence via the random effects, thereby improving statistical inference for the fixed effects if the random effects are independent of the covariates. These models also allows for the estimation of variance within and between the grouping variables included as random effects. In particular, this allows us to separate the school and class effects, which would have been impossible otherwise with the class structure being nested inside schools.

In the following we provide the R code together with the output for the mixed effects model used for the main estimate in our study, the adjusted RR comparing days of absence between HPV vaccinated and unvaccinated girls.

Main_Model <- glmer(data=School_Absence_Data,ABSENCE_ILL~as.factor(HPV)+as.factor(YEAR)+as.factor(GRADE)+

                   as.factor(SEASON)+as.factor(BO)+as.factor(MOTHER_CIV)+as.factor(AGE_AT_BIRTH)+

                   (1|GIRL_ID)+(1|SCHOOL/CLASS_YEAR_COMBO),

                 offset=log(n),nAGQ=0,control=glmerControl(optimizer="nloptwrap"),family='poisson')

summary(Main_Model)

Generalized linear mixed model fit by maximum likelihood (Adaptive Gauss-Hermite Quadrature, nAGQ = 0) ['glmerMod']

Family: poisson  ( log )

Formula: ABSENCE_ILL ~ as.factor(HPV) + as.factor(YEAR) + as.factor(GRADE) +

    as.factor(SEASON) + as.factor(BIRTH_ORDER) + as.factor(MOTHER_CIV) +

as.factor(AGE_AT_BIRTH) + (1 | GIRL_ID) + (1 | SCHOOL/CLASS_YEAR_COMBO)

   Data: School_Absence_Data

Offset: log(n)

Control: glmerControl(optimizer = "nloptwrap")

      AIC       BIC    logLik  deviance  df.resid

 479750.9  480011.4 -239849.4  479698.9    166018

Scaled residuals:

    Min      1Q  Median      3Q     Max

-5.1820 -0.7523 -0.4236  0.3326 17.5530

Random effects:

Groups                     Name        Variance Std.Dev.

GIRL_ID                     (Intercept) 0.76257  0.8733

 CLASS_YEAR_COMBO:SCHOOL (Intercept) 0.23871  0.4886

 SCHOOL                   (Intercept) 0.02682  0.1638

Number of obs: 166044, groups:  GIRL_ID, 14068; CLASS_YEAR_COMBO:SCHOOL, 3543; SCHOOL, 60

Fixed effects:

                                 Estimate Std. Error z value Pr(>|z|)

(Intercept)                     -3.561901   0.076126 -46.790  < 2e-16 ***

as.factor(HPV)1       0.003988   0.010623   0.375 0.707378

as.factor(YEAR)2014             -0.068640   0.012031  -5.705 1.16e-08 ***

as.factor(YEAR)2015             -0.052484   0.018374  -2.856 0.004284 **

as.factor(YEAR)2016              0.019052   0.023775   0.801 0.422933

as.factor(YEAR)2017              0.040243   0.029473   1.365 0.172125

as.factor(YEAR)2018             -0.291170   0.040619  -7.168 7.60e-13 ***

as.factor(GRADE)6            0.003130   0.027661   0.113 0.909904

as.factor(GRADE)7            0.046216   0.029369   1.574 0.115570

as.factor(GRADE)8            0.083424   0.030849   2.704 0.006846 **

as.factor(GRADE)9            0.058560   0.032105   1.824 0.068149 .

as.factor(SEASON)2              -0.495130   0.006406 -77.288  < 2e-16 ***

as.factor(SEASON)3              -0.423573   0.009707 -43.634  < 2e-16 ***

as.factor(SEASON)4              -0.196965   0.009089 -21.670  < 2e-16 ***

as.factor(BIRTH_ORDER)2          0.023901   0.019226   1.243 0.213803

as.factor(BIRTH_ORDER)3         -0.055941   0.028283  -1.978 0.047940 *

as.factor(BIRTH_ORDER)4         -0.070482   0.036919  -1.909 0.056250 .

as.factor(MOTHER_CIV)1        -0.238089   0.016972 -14.029  < 2e-16 ***

as.factor(AGE_AT_BIRTH)(20,25]   0.154825   0.071328   2.171 0.029960 *

as.factor(AGE_AT_BIRTH)(25,30]   0.146941   0.069265   2.121 0.033885 *

as.factor(AGE_AT_BIRTH)(30,35]   0.138277   0.069538   1.989 0.046756 *

as.factor(AGE_AT_BIRTH)(35,40]   0.171192   0.071577   2.392 0.016769 *

as.factor(AGE_AT_BIRTH)(40,100]  0.273884   0.082779   3.309 0.000938 ***

---

Signif. codes:  0 ‘***’ 0.001 ‘**’ 0.01 ‘*’ 0.05 ‘.’ 0.1 ‘ ’ 1

Correlation matrix not shown by default, as p = 23 > 12.

Use print(x, correlation=TRUE)  or

    vcov(x)        if you need it

The Intercept represents the school absence rate for an average girl in an average class in an average school who is HPV unvaccinated, in 2013, in grade 5, in January-March, with no older siblings, and a married mother less than 20 years of age. If we want to calculate the model-based school absence rate for a particular girl in a specific class and a specific school, we need to add 1) the contribution for each fixed effect where the girl is not in the reference category, and 2) the random effects contributions. The random effects contributions are not explicitly presented in the above model output, but we might assume that the particular girl has a 1 SD higher absence rate on the logarithmic scale than the average girl, is from an average class, and from a school with a 1 SD lower absence rate on the logarithmic scale. We might also assume that she is HPV vaccinated but otherwise in all the reference categories for the fixed effects. Thus, we can calculate the absence rate for this particular girl as, exp(-3.562+0.004+0.873-0.1638) = 5.8%. We note that the estimated variances for the random effects reveal that school only has a modest contribution to absence while the individual level provides the greatest contribution. As in the above example, the individual contribution for a girl with 1 SD higher absence rate on the logarithmic scale, corresponds to 2.4 times (exp(0.873)) more absence than the average girl.

**Robustness**

GLMMs are quite data demanding, and thus, when data becomes increasingly sparse, the risks of collinearity, overdispersion and model misspecification increase. We used perturbation analysis to evaluate the robustness of our models of periods of absence and excess days of absence. Perturbation analysis was conducted using the function *manualReclassify* from the R package *perturb*. We reclassified approximately 5% of all the values of schools, grades, calendar years, seasons, birth order, mother’s age at birth, and mother’s civil status, corresponding to slight misclassification of these covariates. We did not reclassify classes due to computational limitations as the computed transition matrix would have been too large. For each model we conducted 50 perturbations resulting in up to 50 simulated vaccine effect estimates (depending on convergence). Due to excessive computation times, we conducted perturbation analysis only on a subset of all the models in our study. We used perturbation analysis on the models of periods of absence of at least 1, 5, 10 and 15 days and on the models of days of absence in excess of 0, 4, 9 and 14 days. The results of these perturbation analyses are presented in Supplementary Figure S1. We notice that models of long absence periods and models of excess days after long periods both give a wider range of estimates and that in particular models of excess days after long periods (9 and 14 days) appear to be volatile. Of the examined models, convergence was not obtained in the analyses of 3 perturbed datasets for illness in excess of 9 days and in the analyses of 16 perturbed datasets for illness in excess of 14 days. We believe that caution is warranted in the interpretation of the vaccine effect estimates from models of excess days of absence after long periods of absence. These data may either be too sparse or be subject to extreme overdispersion.

**Modelling the impact of bias**

We modelled the impact of misclassification of truancy as absence due to illness; we considered the misclassification as non-differential according to vaccination status, and evaluated the impact of 5%, 10% and 20% misclassification of all single days of absence due to illness in the study. Modelling the impact of non-differential misclassification of 5%, 10% and 20% of all single days of absence in the study yielded RRs of 1.00 (95% CI 0.98-1.03), 1.01 (95% CI, 0.98-1.03) and 1.00 (95% CI, 0.98-1.03), respectively.

We modelled how sensitive our data and statistical method was in identifying cause-specific morbidity. Specifically, we simulated the introduction of an association between HPV vaccination and cause-specific morbidity according to different scenarios. Each scenario was defined by a base rate of cause-specific morbidity and a RR representing the association between HPV vaccination and cause-specific morbidity. This was done by sampling a random number from a uniform distribution on the interval [0:1] for each day and converting healthy days to illness-related absence days if the random number assigned to that day was lower than the base rate for the unvaccinated days or the base rate multiplied by the cause-specific morbidity RR for vaccinated days. We assumed that the cause-specific morbidity was uncorrelated with all the underlying covariates. Since the length of absence periods is of no consequence to the main analysis of school days of absence due to illness, we did not model period lengths. In the following table we present the adjusted RRs of absence due to all-cause morbidity estimated under different modelling scenarios. We see that even for base rates of 0.5% (corresponding to an average of a little less than one day of absence due to this illness per year per girl), modest risk ratios would result in statistically significant RRs. Even rarer illnesses could also be detected if the risk ratio was sufficiently large.

| **Cause-specific RR (95% CI) \ base rate** | **0.1%** | **0.2%** | **0.5%** | **1%** | **2%** | **5%** |
| --- | --- | --- | --- | --- | --- | --- |
| **1.25** | 1.01 (0.99-1.03) | 1.02 (1.00-1.04) | 1.03 (1.01-1.05) | 1.05 (1.03-1.07) | 1.08 (1.06-1.10) | 1.13 (1.12-1.14) |
| **1.5** | 1.02 (1.00-1.04) | 1.03 (1.01-1.05) | 1.06 (1.04-1.08) | 1.11 (1.09-1.13) | 1.17 (1.16-1.19) | 1.28 (1.27-1.30) |
| **2** | 1.03 (1.01-1.06) | 1.06 (1.04-1.08) | 1.13 (1.11-1.15) | 1.23 (1.21-1.25) | 1.37 (1.35-1.38) | 1.58 (1.56-1.60) |
| **5** | 1.13 (1.11-1.15) | 1.25 (1.22-1.27) | 1.54 (1.52-1.57) | 1.94 (1.92-1.97) | 2.50 (2.47-2.53) | 3.33 (3.30-3.36) |
| **10** | 1.29 (1.26-1.31) | 1.55 (1.52-1.58) | 2.22 (2.19-2.26) | 3.12 (3.08-3.16) | 4.35 (4.30-4.39) | 6.19 (6.14-6.23) |

**Supplementary Figure S1 Label:**

**Distributions of HPV vaccine effect estimates from perturbation analyses.**

Boxplots of distribution of vaccine effect estimates resulting from the perturbation analyses for selected models of absence period lengths and excess days of absence. The effect estimate based on the real dataset is marked as a colored dot.
