# Supplementary figures and images for: Human papillomavirus vaccination and all-cause morbidity in adolescent girls: a cohort study of absence from school due to illness
Source: Int J Epidemiol. 2021 Feb 6;50(2):518–26. doi: 10.1093/ije/dyab003 (PMC8128452; doi:10.1093/ije/dyab003)

Estimates for the vaccine effect in perturbed datasets

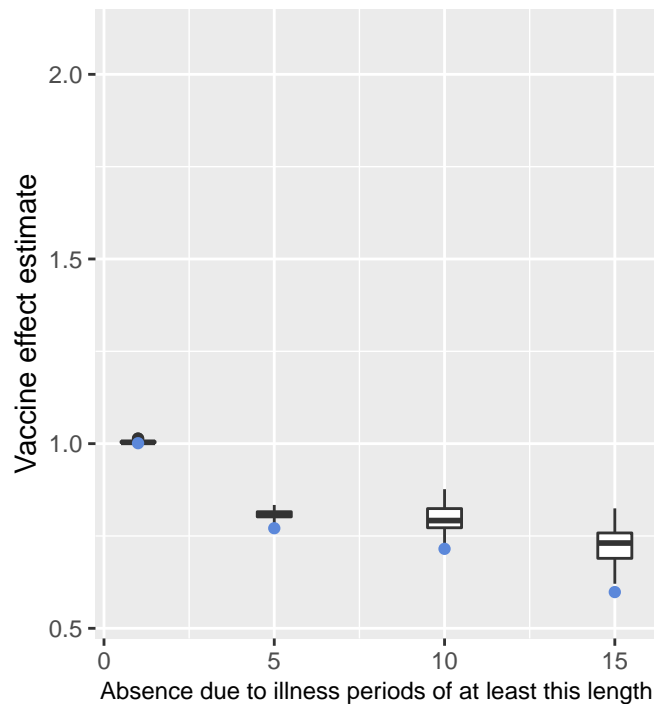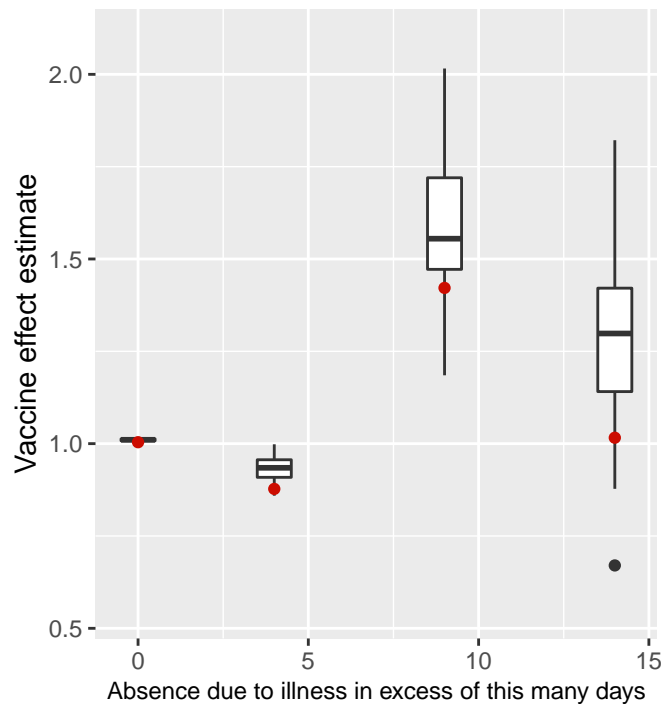

Supplement: dyab003_Supplementary_Data [file dyab003_supplementary_data.zip › ije-2019-12-1617-File006.pdf]
